# The LPG1x family from *Leishmania major* is constituted of rare eukaryotic galactofuranosyltransferases with unprecedented catalytic properties

**DOI:** 10.1038/s41598-018-35847-w

**Published:** 2018-12-04

**Authors:** Jihen Ati, Cyril Colas, Pierre Lafite, Ryan P. Sweeney, Ruixiang Blake Zheng, Todd L. Lowary, Richard Daniellou

**Affiliations:** 1 Institut de Chimie Organique et Analytique, UMR CNRS 7311, Université d’Orléans, Rue de Chartres, BP6759 Orléans, Cedex 02 France; 2 Alberta Glycomics Centre and Department of Chemistry, The University of Alberta, Edmonton, AB T6G 2G2 Canada

## Abstract

Galactofuranosyltransferases are poorly described enzymes despite their crucial role in the virulence and the pathogenicity of numerous microorganisms. These enzymes are considered as potential targets for therapeutic action. In addition to the only well-characterised prokaryotic GlfT2 from *Mycobacterium tuberculosis*, four putative genes in *Leishmania major* were previously described as potential galactofuranosyltransferases. In this study, we have cloned, over-expressed, purified and fully determined the kinetic parameters of these four eukaryotic enzymes, thus demonstrating their unique potency in catalysing the transfer of the galactofuranosyl moiety into acceptors. Their individual promiscuity revealed to be different, as some of them could efficiently use NDP-pyranoses as donor substrates in addition to the natural UDP-galactofuranose. Such results pave the way for the development of chemoenzymatic synthesis of furanosyl-containing glycoconjugates as well as the design of improved drugs against leishmaniasis.

## Introduction

**Figure 1:**
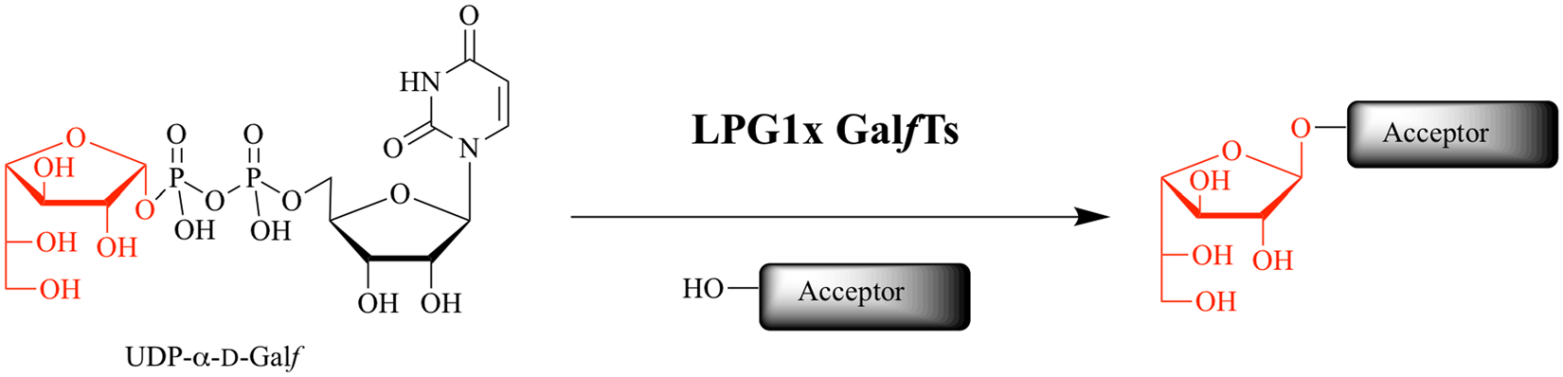
Enzymatic incorporation of a Gal*f* unit from UDP-α-D-Gal*f* onto an acceptor catalysed by a Gal*f*T.

Glycosyltransferases (GTs) (E.C. 2.4.x.x) constitute a large class of enzymes involved in the synthesis of abundant complex glycosidic structures expressed in cells^1^. These glycoconjugates are of upmost importance for the interaction between cells and for infection by pathogenic species^2^. Amongst these GTs, galactofuranosyltransferases (Gal*f*Ts) are poorly described enzymes that catalyse the transfer of a galactofuranosyl moiety (Gal*f*) from UDP-α-D-Gal*f* to specific acceptor molecules (Fig. 1)^3,4^. Noteworthy is that Gal*f* is a five-membered ring sugar that is mainly found on the surface of many pathogenic species such as *Mycobacterium tuberculosis*, *Aspergillus* and *Leishmania* species^5,6^. The peculiarity of this sugar arises from its high immunogenicity^7,8^ and the fact that it is totally absent from mammals^5^. The detection of Gal*f* can therefore be of interest for diagnosis of infection^9,10^. Even more interestingly, as it can be classified as a virulence factor, the inhibition of the biosynthesis of Gal*f*-containing conjugates can lead to the development of promising inhibitors from which may arise powerful drugs against Multi-Drug-Resistant tuberculosis, for example^11,12^. The unique structure of Gal*f* in the glycosciences has therefore drawn the attention of many researchers due to its potential tremendous therapeutic applications.

Despite this interest, very few Gal*f*Ts have been studied to date. The most investigated microbial organism expressing Gal*f*T is *M. tuberculosis*, whose Gal*f*Ts involved in cell wall arabinogalactan biosynthesis have been cloned and characterized^13–15^. In the galactan chain, Gal*f* is alternatively bound by β-(1 → 5) and β-(1 → 6) glycosidic bonds. The two Gal*f*Ts involved in galactan chain initiation and elongation are bifunctional, and are able to catalyse the formation of two different glycosidic bonds. Besides those two, much less information is known on WbbI from *Klebsiella pneumoniae*^16^, which is another bifunctional Gal*f*T, and on GfsA from Aspergillus^17^. In *Trypanosoma rangeli*, genes were identified as coding for Gal*f*T, however, the enzymatic activity of the corresponding protein could not be demonstrated^18^.

Leishmaniasis belongs to the group of Neglected Tropical Diseases, as defined by World Health Organization (WHO), which includes diseases that are endemic to Third World countries. More than 20 species of parasites are responsible for the yearly infection of two million people, threatening about 350 million people worldwide. It is estimated that more than 12 million people are infected by the parasite in nearly 100 countries^19^. This disease can exhibit several clinical forms, from the common cutaneous form, which is generally a self-healing disease, to the visceral form (Kala-azar) that is the most severe, usually fatal manifestation of *Leishmania* infection. Moreover, leishmaniasis has emerged as one of the most important opportunistic infection associated with HIV. In southern Europe, 70% of visceral leishmaniasis are associated with HIV infection^20^. For these reasons, leishmaniasis is considered as one of the most fatal Neglected Tropical Disease, and has drawn WHO’s attention concerning its diagnosis and treatment^21^. Efficient prophylactic measures, including safe vaccines, are not available, and effective and affordable chemotherapy is lacking. Current treatments that rely on toxic antimony-containing compounds or diamidines, require strict medical supervision and are threatened by the spread of drug resistance^22^. Other medications such as amphotericin B or miltefosine offer an alternative for treatment but are also toxic and expensive. In addition, the emergence of resistant strains is expected for miltefosine because of its long half-life^23^. There is thus an urgent need to identify new chemotherapeutic agents for the treatment of this disease.

**Figure 2:**
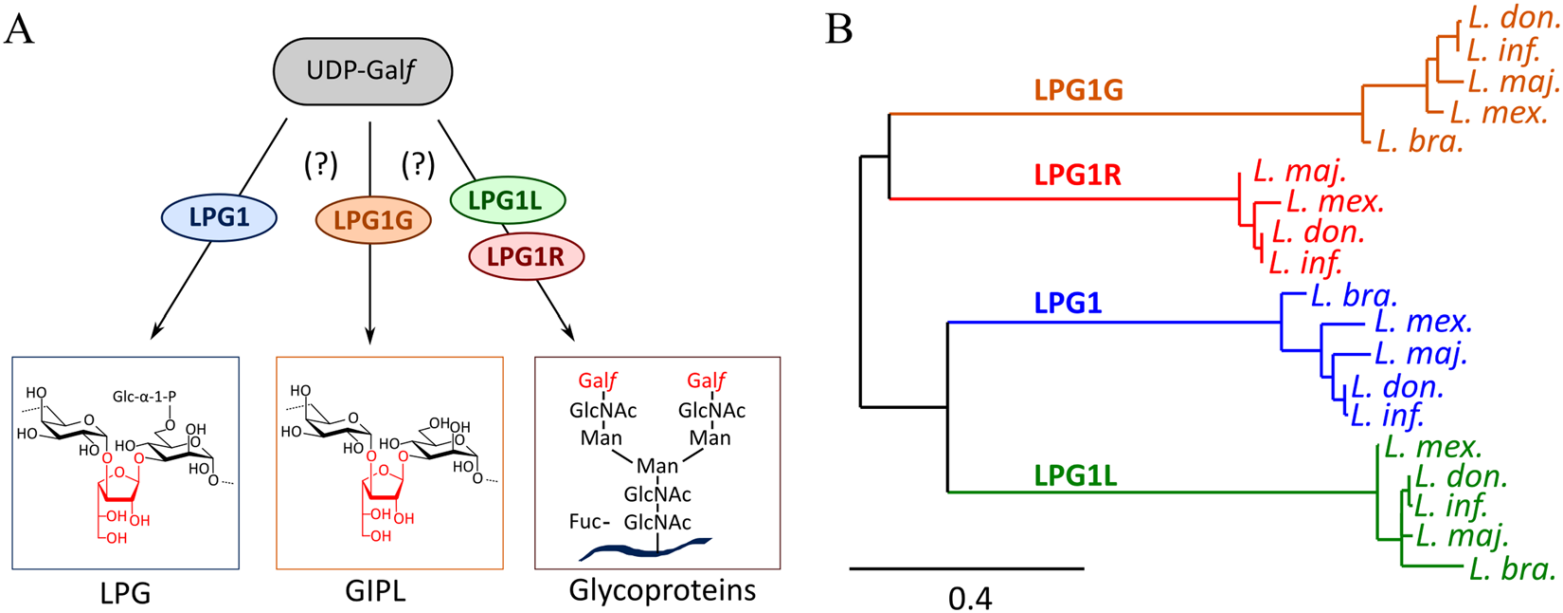
(**A**) Putative biological roles of *Leishmania major* Gal*f*Ts. (**B**) Phylogeny of Gal*f*Ts between main pathological *Leishmania* species (*L. major, L. infantum, L. donovani, L. mexicana, and L. braziliensis*). The bottom bar scales the genetic change (*ie* ratio of substitutions per site) along horizontal branches. Starting from a MUSCLE alignment of LPG1, LPG1G, LPG1L, and LPG1R sequences from TriTrypDB database in *L. major* (resp. LmjF.25.0010, LmjF.32.3990, LmjF.26.0550, LmjF.33.0300), *L. infantum* (LinJ.25.0010, LinJ.32.4140, LinJ.26.0520, LinJ.33.0330), *L. donovani* (LdBPK_250010.1, LdBPK_324140.1, LdBPK_260520.1, LdBPK_330330.1), *L. mexicana* (LmxM.25.0010, LmxM.31.3990, LmxM.26.0550, LmxM.32.0300), and *L. braziliensis* (LbrM.25.0010, LbrM.32.4230, LbrM.26.0650), the tree was built using PhyML software using Neighbourg Joining algorithm in the Phylogeny.fr web server^37^.

The discovery of new antiparasitic compounds exhibiting low toxicity and high specificity for *Leishmania* relies on the identification of new therapeutic targets. The first pyranose–furanose mutase from an eukaryotic organism has been recently characterized in *Leishmania*^24^. Targeted gene deletion of this enzyme in *Leishmania major* led to attenuated virulence, establishing that Gal*f* contributes significantly to *L. major* pathogenesis^7^. Investigations on Gal*f*T’s have started with expression and isolation of the *M. tuberculosis* enzymes^25^. The nature of the reactions catalyzed (processing transferases) and the structure of the Gal*f*-containing glycans in *M. tuberculosis* are very different from the *Leishmania* system. However, studies of *M. tuberculosis* Gal*f*Ts have provided data relevant for the design of antituberculosis agents^25,26^. In Leishmania, galactofuranose-containing glycoconjugates such as lipophosphoglycans (LPGs), glycosylinositolphospholipids (GIPLs) and glycoproteins were reported as playing important role in parasite infection process (Fig. 2)^27–30^. Moreover all these structures have important functions in the parasite lifecycle^31^. They play a key role in growth and communication between the parasite and mammalian cells^32^. In addition, they are essential for the binding and detachment of the parasite from the midgut of the insect vector and therefore for the transmission of the parasite to the mammalian host^33–35^. They also confer resistance to complement-mediated lysis and inhibit phagolysosomal fusion^36^. Therefore, to permit a better understanding of the implication of each Gal*f*-containing glycoconjugates in cell wall pathogenesis, it is important to identify and characterize the Gal*f*Ts that are involved in their assembly^37^.

The genome of *Leishmania major* was previously screened by Zhang and co-workers, and an analysis led to the identification of four putative genes (*lpg1, lpg1L, lpg1R, and lpg1G*) that could encode for Gal*f*Ts, but that share less than 20% similarity of sequence with known Gal*f*Ts from other species^30,38^. Knock-out studies enabled the authors to identify the enzyme LPG1 as the Gal*f*T involved in Gal*f* attachment during LPG biosynthesis^39^. The generation of knock-out mutants enabled subsequent studies to identify LPG as necessary for insect infection, as well as critical for parasite virulence and survival in the early stages of the human infection, although some controversy still remains depending on the species^31,39,40^. Deletion of *lpg1, lpg1L, or lpg1R* (single or multiple deletions) helped the authors to attribute respective functional roles of these three enzymes (Fig. 2A)^30^, although no information on LPG1G nor study of any of the four proteins at a molecular level was available. Moreover, homologous enzymes are also present in other *Leishmania* species (*infantum*, *donovani*, *braziliensis*, *mexicana*) (Fig. 2B). LPG1R is only present as a truncated protein in *L. braziliensis*, thus the protein was not used for alignment and tree generation. All four proteins share 52–92% homology (27–30% identity) with *L. major* orthologs. Studying these Gal*f*Ts is therefore crucial to understand the chemistry of the Gal*f*T enzymatic reaction, and their biological role in *L. major*. Moreover, galactofuranose residues were also detected in glycoconjugates of *Trypanosma* species^5^; however, only genes coding for putative galactofuranosyltransferases were described in *T. rangeli* and in *T. cruzi*^18,41^. Biochemical tools and knowledge of these enzymes developed in *L. major* could thus serve as a template for other pathogenic systems, especially trypanosomatids for which no satisfactory treatments are available to date. Here we report the first cloning, overexpression, purification and biochemical characterization of these four proteins from *L. major* as well as the identification of their enzymatic function.

## Material and Methods

### Chemical and biological reagents

Chemical reagents including buffer, salts, sugars, NAD^+^, pyruvate kinase and lactic dehydrogenase enzymes from rabbit muscle were purchased from Sigma-Aldrich. UDP-pyranoses and pNP-sugars were purchased from Carbosynth (Compton, UK), pET-vectors from Novagen and pMal vectors from New England Biolabs. UDP-α-D-Gal*f* was enzymatically prepared, purified and characterized following the procedure previously developed by Prof. Field’s team^42^.

### Cloning and expression of the four putatives genes

The four genes *lpg1, lpg1L, lpg1R* and *lpg1G* were amplified by PCR using *L. major* genomic DNA, which was kindly provided by Dr Françoise Routier (Hannover Medical School), as template. Specific primers described in Table S1 were designed to amplify genes that encode for proteins without the transmembrane domain (according to TMHMM prediction server)^43^. *lpg1*, *lpg1L* and *lpg1R* loci (TritrypDB Accession numbers LmjF.25.0010, LmjF.26.0550, LmjF.33.0300), and the 3 identical lpg1G genes copies located in 3 distinctive loci (TritrypDB Accession LmjF.32.3990, LmjF.05.1230, LmjF.19.1650) were used as template for primer design. The amplified region for *lpg1*, *lpg1L*, *lpg1R* and *lpg1G* excluded the transmembrane domains (resp. nucleotides 1–117, 1–120, 1–120, and 1–139). Amplicons were cloned into pMAL-c2X vector to generate *lpg1*-pMAL, *lpg1G*-pMAL, *lpg1L*-pMal, and *lpg1R*-pMal plasmids (Figs S1–S4). The sequencing of the DNA performed by Eurofins Genomics validated the cloned constructs. The corresponding MBP-fused protein thus contained respectively residues 40–396, 41–421, 41–592, and 47–599.

Plasmids were transformed into *E. coli* Rosetta (DE3) strain. Clones were cultivated on LB Broth medium with the appropriate antibiotics (Chloramphenicol 30 μg/mL and Ampicillin 50 μg/mL) at 37 °C until optical density at 600 nm reached 0.6. Overexpression was then induced with 100 µM of IPTG and the cultures were incubated overnight at 30 °C. Two liters of culture were harvested and resuspended in Tris 50 mM pH 8.0 Buffer containing NaCl 25 mM and 1 mg/mL of lysozyme. Resuspension was then incubated for 30 min with stirring at 4 °C before freeze–thaw lysis, followed by sonication. After centrifugation (40000 g, 20 min, 4 °C), the supernatant was filtered and loaded on Maltose Binding Protein (MBP) affinity column (MBP Trap HP-1mL, GE Healthcare), and MBP-tagged proteins were then eluted according to manufacturer instructions. LPG1x recombinant proteins were finally purified by size exclusion chromatography (SuperdexTM 200 10/300 GL, GE Healthcare). The proteins of interest were considered as pure enough (>95%) according to SDS-PAGE to perform enzymatic assays. The concentration of contaminants was too low to enable their identification by mass fingerprinting after gel excision. The molecular weight, purity, and concentration were assayed by respectively MS, SDS-PAGE, and the Bradford assay^44^.

### Coupled spectrophotometric assay

Enzymatic assays were performed in 96-well microtiter plate following protocol previously described^45^. A few parameters were modified: the final volume of the reaction was 200 µL and the media contained 0.2–10 µg of enzyme. Commercially available acceptors were tested at a final concentration of 1 mM. Twenty-nine commercially available carbohydrates were tested as acceptors including hexoses (D-Glc, D-Man, D-Glc*N*H_2_, D-Glc*N*Ac), monosaccharides (Me-α-D-Glc, Me-α-D-Man and Oct-α-D-Man), *p*NP-furanoses (*p*NP-α-L-Ara*f*, *p*NP-β-D-Gal*f* and *p*NP-β-D-Rib*f*), *p*NP-hexoses (*p*NP-α- and β-D-Glc, *p*NP-α- and β-D-Gal, *p*NP-α- and β-D-Man, *p*NP-α- and β-D-Glc*N*ac, *p*NP-α- and β-D-Xyl, *p*NP-α- and β-L-Ara, *p*NP-α- and β-L-Fuc, *p*NP-β-D-Fuc and *p*NP-α-L-Rha) and disaccharides (D-Maltose, D-Lactose and D-Melibiose). Controls *i.e*. reaction’s mixture missing the donor, the acceptor or the enzyme, were performed in parallel. The reactions were monitored at 340 nm using a Multiskan™ GO (Thermo Scientific) microplate reader for up to 20 min with 10 s intervals. UDP formation rates were assumed to be equal to NADH consumption rates, and kinetic parameters were calculated by fitting saturation curves (obtained from the average of triplicate measurements) with standard the Michaelis–Menten equation (Eq. 1)^46^, using Prism 6 (GraphPad) (see Fig. S5).

1 $${v}_{0}=\frac{{V}_{{\rm{\max }}}\times [S]}{{K}_{{\rm{M}}}+[S]}$$

Michaelis–Menten equation. [S] is the substrate concentration, *v*_0_ and *V*_max_ are, respectively, the initial and the apparent maximum velocity rate, and *K*_M_ is the apparent Michaelis constant.

### Glycosylation reaction assay and High-Resolution Mass Spectrometry (HRMS) analysis

A magnetically stirred 3 mL solution containing methyl α-D-mannopyranoside (25 μmol, 5 mg), the UDP-sugar (5 μmol, 2.8 mg), 50 mM Tris pH 8, 20 mM MgCl_2_ and 0.5 mg of the Gal*f*T to be tested was prepared. The reaction was incubated at 37 °C for 24 h. Analytical thin layer chromatography of the reaction mixture was performed on silica gel aluminium supported plates. The eluent was composed of ethyl acetate, methanol and water with the ratio of 7/2/1. Sugars were detected with orcinol solution (95% ethanol 100%, 5% H_2_SO_4_ and orcinol 200 mg) after heating at 100 °C (see Fig. S6). After reaction solvent evaporation, 1 mL of acetic anhydride and 1 mL of pyridine were added and the reaction was left at room temperature for 48 h. Then, the residue was concentrated through co-evaporation with toluene. The reaction mixture was then resuspended and the peracetylated sugar was isolated by extraction into CH_2_Cl_2_. Finally, high-resolution accurate mass measurements were performed in positive mode with an ESI source on a Q-TOF mass spectrometer (Bruker MaXis) with an accuracy tolerance of 2 ppm by the “Fédération de Recherche” ICOA/CBM (FR2708) analytical platform (see Fig. S7).

## Results and Discussion

### Expression of 4 *lpg1X* family genes as recombinant soluble proteins

**Figure 3:**
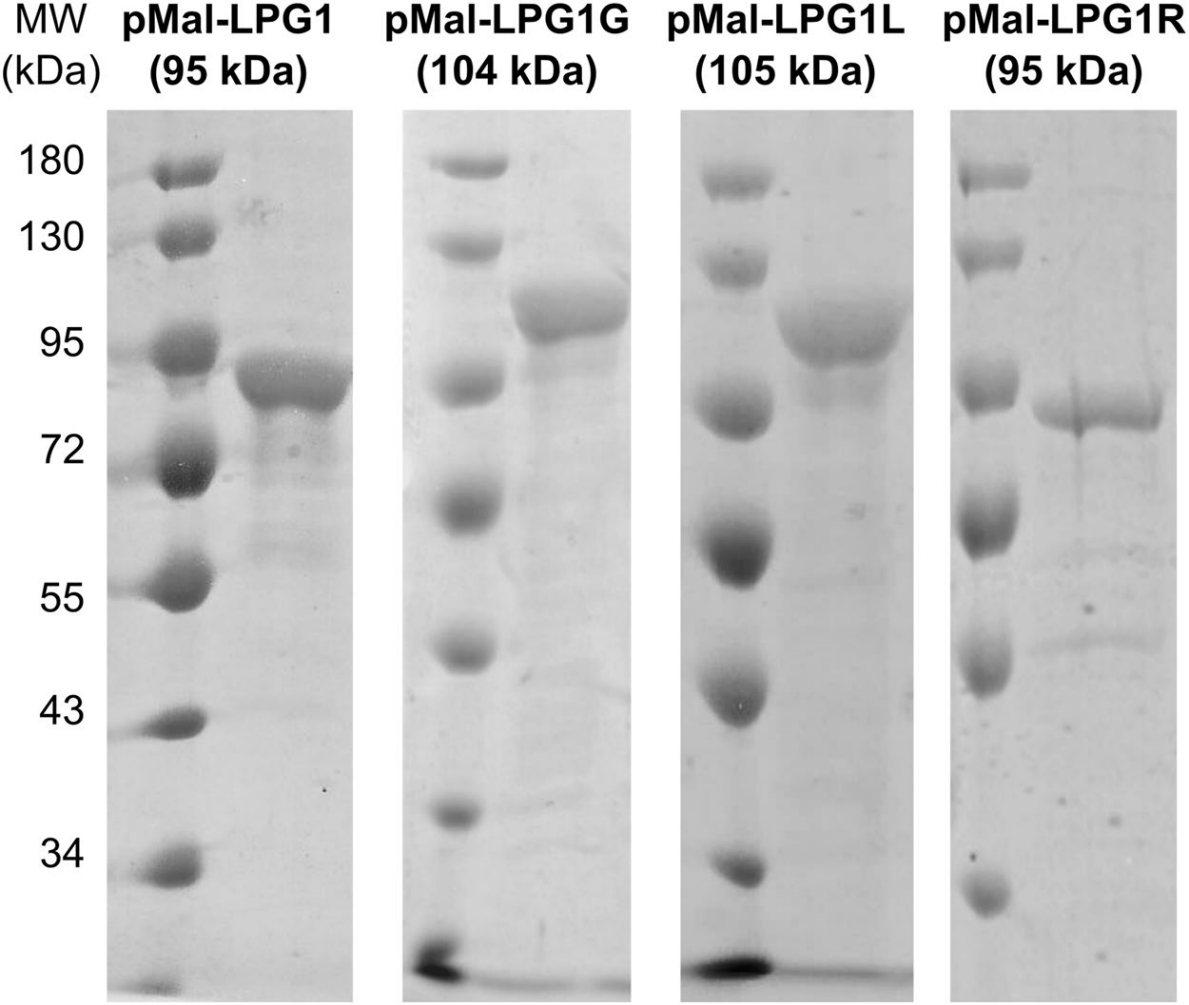
Evaluation of the expression and the purity of *L. major* Gal*f*Ts after superdex elution step in 1-D 8% SDS-PAGE with standard mixture marker proteins. Full gels are displayed in SI in Figs S2–5c.

The genes *lpg1, lpg1L, lpg1R and lpg1G* were amplified by PCR from *Leishmania major* genomic DNA, using primers designed to remove the *N*-terminal transmembrane domain. Interestingly, unlike other *lpg1X* genes, *lpg1G* has a particular genetic context. In *L. major* genome, three identical copies of *lpg1G* gene are found in three distinct loci, all located near the telomeric end of the corresponding chromosomes. An increase of the copy number of genes near the telomeric end of chromosomes in *Leishmania* has been related to drug resistance mechanisms^47,48^. However, in the case of *lpg1G gene*, the copy numbers are located in separate chromosomes, making this gene a unique example for which the biological function and significance of amplification has still to be understood. For all 4 genes, the amplified fragments were initially cloned into different pET expression vectors (containing *N*- or both *N*- and *C*-terminal His-tag) such as pET-24b(+), pET-28a(+) and pET-32a(+) and transformed in various strains of *E. coli*. Each His-tagged recombinant protein was over-expressed and purified but the obtained proteins were very difficult to purify, as they formed strong complexes with the GroEL chaperonin (as identified by HRMS) and very low yields were obtained. Different literature protocols were tested to remove the contaminant but none was successful^49^. Thus, in our hands, pET vectors were found not suitable for expressing the LPG1x proteins. To overcome this issue, the *lpg1X* genes were cloned in the pMAL-c2X expression vector to obtain MBP-tagged proteins. Constructs were transformed into *E. coli* Rosetta (DE3) expression strain and proteins were over-expressed at 30 °C. Chemical and physical lysing technics using lysozyme, heat shock and sonication were performed, followed by affinity and size exclusion chromatography. Finally, the pure desired recombinant proteins (Fig. 3) were obtained with a high yield of 5 mg/L for LPG1 and LPG1R and 10 mg/L of culture for LPG1G and LPG1L.

### Enzymatic assays

We used a coupled spectrophotometic assay to assess Gal*f*T activity^45^. This assay correlates the formation of UDP with NADH consumption by coupling the activity of Gal*f*T to two enzymes, pyruvate kinase (PK) and lactate dehydrogenase (LDH). On the twenty-nine commercially available carbohydrates that were tested as acceptors, including hexoses, monosaccharides, *p*NP-furanoses, *p*NP-hexoses and disaccharides, only methyl α-D-mannopyrannoside (Me-Man*p*) efficiently reacted as an acceptor when used at 1 mM. It was therefore used as a simple acceptor, instead of synthesizing the complex natural acceptor. The acceptor ability of Me-Man*p* was anticipated, as a Gal*f*–Man*p* linkage is present in both LPG and GIPL (see Fig. 2).

### LPG1, LPG1L, LPG1R and LPG1G are galactofuranosyltransferases

Using Me-Man*p* as the acceptor, kinetics data was obtained (three replicate experiments) with the respective enzymes. Michaelis–Menten analysis of the data is shown in Table 1. All four enzymes recognized UDP-Gal*f* with apparent *K*_M_ values ranging from 0.02 to 0.55 mM. Based on that parameter, the proteins can be clustered in two groups: LPG1, LPG1G, and LPG1L, which strongly bind to UDP-Gal*f* with *K*_M_’s in the low μM level, and LPG1R, which binds UDP-Gal*f* more weakly. The UDP-Gal*f K*_M_ value for LPG1R is comparable to the one reported for mycobacterial GlfT2 (0.38 mM). Moreover, the *k*_cat_ values of LPG1, LPG1G and LPG1L range from 5,000 to 30,000 min^−1^, which are superior to the catalytic rate for LPG1R, which is at least 10-fold time lower with a value of 636 min^−1^ (and comparable to the catalytic rate reported for GlfT2). The low enzymatic activity observed for LPG1R, in comparison with the three other Gal*f*Ts might be explained by uncorrected folding of the enzyme, because of the removal of *N*-terminal transmembrane domain. However, this hypothesis could be assessed, as for all other LPG1x expressed proteins, no loss of activity could be observed. Moreover, as all four proteins were soluble and no preliminary structural data (*eg*. Circular Dichroism) on full-length protein is available, validation of proper folding could not be confirmed. These marked differences in the Michaelis constant and turnover rate are found also when estimating the catalytic efficiency (*k*_cat_/*K*_M_) of LPG1, LPG1G and LPG1L yielding values higher than 300,000 min^−1^ mM^−1^ as opposed as 1,145 min^−1^ mM^−1^ and 1,131 min^−1^ mM^−1^ for LPG1R and GlfT2 respectively. In summary LPG1, LPG1G and LPG1L demonstrated strong *in vitro* Gal*f*Ts properties, at least 300 times higher than LPG1R and the previously reported mycobacterial GlfT2 from *M. tuberculosis*. However, in *M. tuberculosis* GlfT2 is a polymerizing enzyme that adds around 30 Gal*f* units, linked by alternating by β-(1 → 5) and β-(1 → 6) glycosidic bonds^50^. GlfT2 belongs to the CAZY glycosyltransferase (GT) family 2^51,52^, which contains mainly polymerizing enzymes such as the cellulose or the chitin synthase. On the contrary, the LPG1x enzymes belong to the CAZY GT family 40, which only contains putative Gal*f*Ts from trypanosomatids. In *Leishmania* species, Gal*f*Ts introduce only one Gal*f* residue into the 3-OH position of the mannosyl acceptor with β-selectivity for example in LPG and GIPL (see Fig. 2).

**Table 1:** Kinetic parameters of leishmanial Gal*f*Ts LPG1, LPG1G, LPG1L, LPG1R compared with mycobacterial GlfT2 for UDP-α-D-Gal*f*.

	Enzyme	Apparent *K*_*M*_^a^ (mM)	*k*_*cat*_^a^ (min^−1^)	*k*_*cat*_*/K*_*M*_^a^ (min^−1^ mM^−1^)
UDP Gal*f*	LPG1	0.07 ± 0.07	30,750 ± 62	410,000
	LPG1G	0.03 ± 0.04	12,352 ± 58	393,655
	LPG1L	0.02 ± 0.02	5,296 ± 63	290,800
	LPG1R	0.55 ± 0.68	636 ± 76	1,145
	GlfT2^14^	0.38 ± 0.06	430 ± 35	1,131

^a^Me-Man*p* was used as the acceptor.

In addition, after subsequent peracetylation of the mixture following concentration, HRMS analysis demonstrated the presence of the corresponding disaccharide, indicated by the presence of a peak at *m/z* = 673.1950 corresponding to the exact mass of the sodium adducts of the peracetylated and glycosylated methyl α-D-mannopyranoside product (see Fig. S7). Unfortunately ^1^H, HMBC or HMQC NMR experiments lead to weak signals, probably due i) to the low amount of dissacharide or most probably, ii) to the presence of a mixture of (1–2, 1–3, 1–4 and/or 1–6) regioisomers. It is noteworthy that Gal*f*-man*p* containing structures can also be found in other pathogenic microorganisms such as *Cryphonectria parasitica* (1–2 linkage), *Aspergillus* (1–3 or 1–6 linkages) or *Paraccidioides brasiliensis* (1–6 linkage)^5^. Still, this HMRS data unambiguously confirms that the LPG1x family can catalyse the transfer of a Gal*f* residue to Me-Man*p*. These Gal*f*T activities are unique both (i) in term of their high catalytic efficiency toward the UDP-Gal*f* and (ii) because they are the first, and to date only, kinetically characterized enzymes from the CAZY GT family 40. Specially given their high turnover values, comparable to those of sucrose or glycogen phosphorylases, and although it will require more studies to discover efficient acceptors for these enzymes, they constitute original biocatalytic tools that will be useful for the chemoenzymatic synthesis of galactofuranosyl-containing conjugates. Such compounds are expected to be useful biological probes for studying cytosolic mutases or eukaryotic transporters^53–55^ present in the Golgi membranes of *Leishmania*.

### LPG1x family can also use UDP-pyranoses as sugar donors

Five NDP-pyranoses were also tested with each LPG1x Gal*f*T (Table 2) so to probe the substrate specificity of these enzymes with artificial donors. Unexpectedly, all four of the enzymes were able to use UDP-pyranoses as donor substrates. Preparative reactions were incubated at 37 °C for 24 h and the reactions were followed by TLC (see Fig. S6). The product of these reactions exhibited an R_f_ = 0.16, similar to the maltose used as a reference. HRMS analysis of the peracetylated sugar products enabled the identification of the corresponding disaccharide (see Fig. S7). None of the four-recombinant proteins was able to use UDP α-D-glucuronic acid as a substrate. LPG1, LPG1G and LPG1R were the less promiscuous as they were only able to recognize only one UDP-pyranose. LPG1 and LPG1G recognize UDP-α-D-Gal*p* with respective *k*_cat_/*K*_M_ values of 1,400 min^−1^ mM^−1^ and 27,978 min^−1^ mM^−1^. This is far lower than for the UDP-Gal*f* and this is mainly due to a much lower *k*_cat_. Indeed, the apparent *K*_M_ is still in the sub-mM range, even as low as 5 μM for LPG1G. LPG1R only recognized UDP-α-D-Glc*p* a similar kinetic properties to the two previous Gal*f*Ts for the UDP-Gal*p*, *i.e*. a low *k*_cat_/*K*_M_ (7,994 min^−1^ mM^−1^). Still this is close to a seven-fold increase as compared to UDP-Gal*f* and therefore LPG1R exhibits a better glucopyranosyltransferase than galactofuranosyltransferase activity at least *in vitro*. LPG1L was the most promiscuous enzyme in this respect as it was able to catalyse the reaction with not only UDP-α-D-Gal*p* and UDP-α-D-Glc*p* but also very surprisingly with GDP-α-D-Man*p* and GDP-α-D-Glc*p* even with lower specificity. Once again, with LPG1L Gal*f*T the apparent *K*_M_ values were in the tens of millimolar range and the *k*_cat_ values were as low as 24 min^−1^. NDP-pyranoses were all recognized in a similar manner but UDP nucleotide sugars led to faster reactions than their GDP counterparts by 50 to 100-fold. Among the few characterized Gal*f*Ts, only *M. tuberculosis* Gl*f*T2 has been reported to be able to use and incorporate analogues of galactofuranose (deoxy and fluoro derivatives)^26^. However, LPG1x exhibit higher substrate promiscuity, as they can utilize UDP-pyranose donors.

**Table 2:** Kinetic values of LPG1, LPG1G, LPG1L and LPG1R for UDP-D-pyranoses (n.d: no enzymatic activity detected).

	UDP-pyranose	Apparent *K*_*M*_^a^ (mM)	*k*_*cat*_^a^ (min^−1^)	*k*_*cat*_*/K*_*M*_^a^ (min^−1^ mM^−1^)
LPG1	UDP α-D-Gal*p*	0.23 ± 0.02	320 ± 22	1,400
	UDP α-D-Glc*p*	n.d	n.d	n.d
	UDP α-D-GlcA	n.d	n.d	n.d
	GDP α-D-Glc*p*	n.d	n.d	n.d
	GDP α-D-Man*p*	n.d	n.d	n.d
LPG1G	UDPα-D-Gal*p*	0.005 ± 0.001	132 ± 10	27,978
	UDP α-D-Glc*p*	n.d	n.d	n.d
	UDP α-D-GlcA	n.d	n.d	n.d
	GDP α-D-Glc*p*	n.d	n.d	n.d
	GDP α-D-Man*p*	n.d	n.d	n.d
LPG1L	UDPα-D-Gal*p*	0.05 ± 0.01	468 ± 50	9,900
	UDP α-D-Glc*p*	0.006 ± 0.001	205 ± 26	34,700
	UDP α-D-GlcA	n.d	n.d	n.d
	GDP α-D-Glc*p*	0.043 ± 0.005	24 ± 3	500
	GDP α-D-Man*p*	0.032 ± 0.005	86 ± 6	2,860
LPG1R	UDPα-D-Gal*p*	n.d	n.d	n.d
	UDP α-D-Glc*p*	0.038 ± 0.005	303 ± 33	7,994
	UDP α-D-GlcA	n.d	n.d	n.d
	GDP α-D-Glc*p*	n.d	n.d	n.d
	GDP α-D-Man*p*	n.d	n.d	n.d

^a^Me-Man*p* was used as the acceptor.

The ability of LPG1L to recognize and utilize a diversity of nucleotide-sugar donors places this enzyme among the most promiscuous natural and characterized glycosyltransferase reported to date in term of the donor. This finding also underscores this protein as a promising tool for glycorandomization, at least for transferring carbohydrate residues to α-Man*p*-containing acceptors^56,57^. The hypothesis that this activity results from the residual activity of a contaminant mutase from *E. coli* followed by classical Gal*f*T activity was ruled out for three reasons: (i) UDP-α-D-Glc*p*, GDP-α-D-Man*p* and GDP-α-D-Glc*p* are not reported substrates of the mutase^58^, (ii) the presence of a residual band at 42 kDa on SDS-PAGE corresponding to the mass of the mutase was not observed (see Fig. 3)^59^, and (iii) the obtained kinetic parameters are incompatible with those observed for the mutase as a contaminant^60^.

## Conclusion

Despite the natural occurrence of the galactofuranose in many pathogenic microorganisms, the pathways involved in its biosynthesis remain poorly understood. This is due, in part, to a lack of knowledge of the corresponding enzymes involved in its incorporation into glycoconjugates (mutases, transferases, transporters, hydrolases). The tedious synthesis of the required donor substrate, UDP-α-D-Gal*f*, is another barrier^4,61,62^. This work provides the first enzymatic characterization of eukaryotic Gal*f*Ts and substantially increases our knowledge of these rare enzymes from the CAZY GT family 40. In addition to UDP-α-D-Gal*f*, these enzymes proved *in vitro* to be able to use some NDP-pyranoses as substrates, thus indicated that they are among the most promiscuous natural glycosyltransferases to date. These unique biocatalysts also proved to be stable and robust for days and can now serve for the chemo-enzymatic incorporation of Gal*f* moiety into complex glycoconjugates.

## Electronic supplementary material

Supplementary Information
