# Supplementary material for: The LPG1x family from Leishmania major is constituted of rare eukaryotic galactofuranosyltransferases with unprecedented catalytic properties
Source: Sci Rep. 2018 Dec 4;8:17566. doi: 10.1038/s41598-018-35847-w (PMC6279836; doi:10.1038/s41598-018-35847-w)
Supplement: Supplementary file 1 — Supplementary Information [file 41598_2018_35847_MOESM1_ESM.docx]

**The LPG1x family from *Leishmania major* is constituted of rare eukaryotic galactofuranosyltransferases with unprecedented catalytic properties**

Jihen Ati,^1^ Cyril Colas,^1^ Pierre Lafite,^1^ Ryan P. Sweeney,^2^ Ruixiang Blake Zheng,^2^ Todd L. Lowary^2^ and Richard Daniellou^1,^*

^1^ Institut de Chimie Organique et Analytique, UMR CNRS 7311, Université d’Orléans, Rue de Chartres, BP6759, Orléans Cedex 02, France.

^2^ Alberta Glycomics Centre and Department of Chemistry, The University of Alberta, Edmonton, Canada, AB T6G 2G2.

**Contact:** [richard.daniellou@univ-orleans.fr](mailto:richard.daniellou@univ-orleans.fr)

Table S1 Primers sequences used Page 1

Figure S1 LPG1G construct and protein Page 2

Figure S2 LPG1L construct and protein Page 3

Figure S3 LPG1R construct and protein Page 4

Figure S4 LPG1 construct and protein Page 5

Figure S5 Michaelis–Menten plots Page 6–7

Figure S6 Typical TLC plate Page 8

Figure S7 HRMS Page 9

Table S1: Primers sequences used to clone *lpg*1, *lpg*1L, *lpg*1R and *lpg*1G into pMal-c2X vector. Corresponding restriction sites added are highlighted in italic.

| Gene | Primers | | Restriction enzyme |
| --- | --- | --- | --- |
| *lpg1* | Forward | TT*GGATCC*CGCTCGGGCACAGAGACCT | BamHI |
|  | Reverse | GG*AAGCTT*TTAGCTAGGATCAACAGCAAAG | HindIII |
| *lpg1L* | Forward | TT*GGATCC*GACGTAACACAGCCCAC | BamHI |
|  | Reverse | TTT*CTAGAT*TAAGGGCTGACAGCCTGCA | XbaI |
| *lpg1R* | Forward | TT*GGATCC*GACGCGGCGGTGGGAG | BamHI |
|  | Reverse | TT*AAGCTT*CTACTTTCGCCAATCCGGCTC | HindIII |
| *lpg1G* | Forward | TT*GAATTC*GCGCTCGGATGGACAAG | BamHI |
|  | Reverse | TT*AAGCTT*TCATTGGTAAGCAAAC | HindIII |

Figure S1a: *lpg1G*-pMal plasmid construct


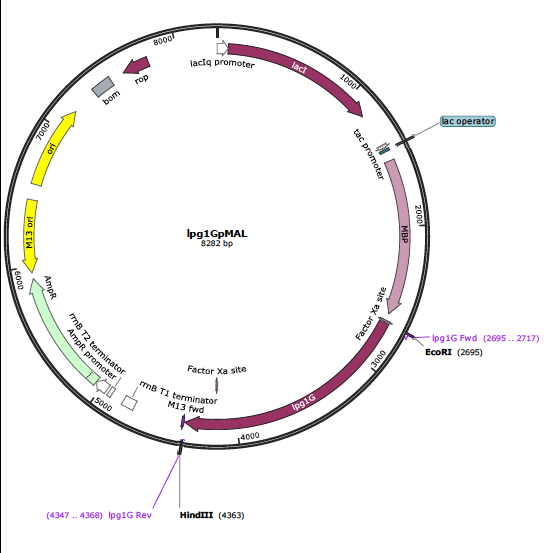


Figure S1b: LPG1G-pMal protein


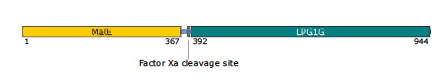


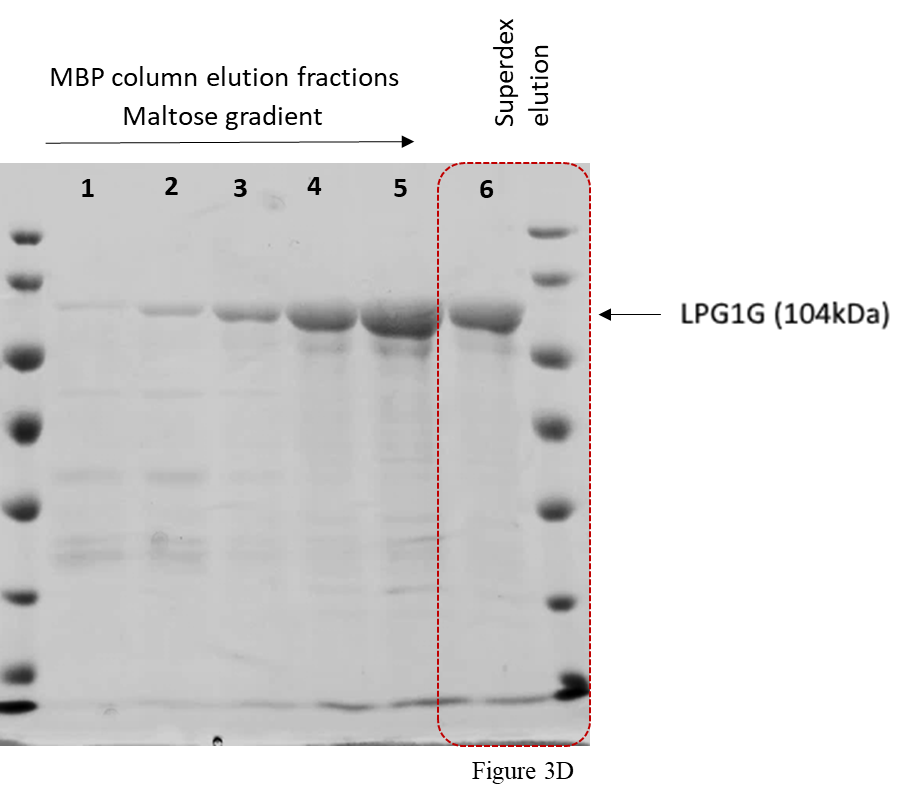


Figure S1c: Evaluation of the expression and the purity of *L. major* LPG1G (104kDa) in 1-D 8% SDS-PAGE with standard mixture marker proteins. Lane 1-5: Elution fractions after MBP column purification step with a maltose gradient. Lane 6: Elution fraction after superdex elution step. The part of the gel, which is shown in Figure 3, is highlighted in red dashed box.

Figure S2a: *lpg1L*-pMal plasmid construct


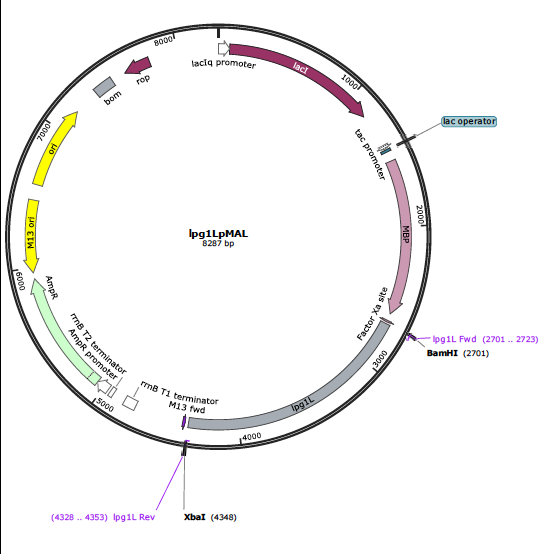


Figure S2b: LPG1L-pMal protein


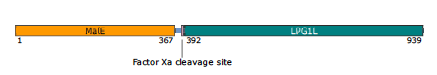


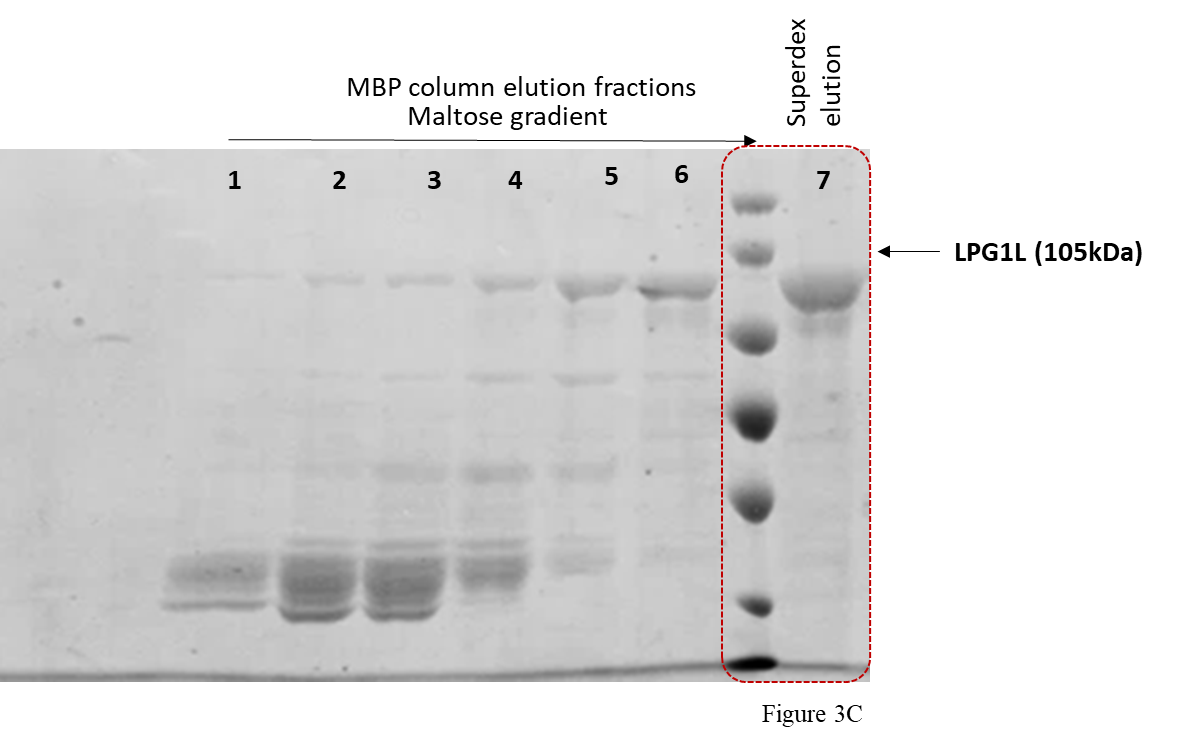


Figure S2c: Evaluation of the expression and the purity of *L. major* LPG1L (105kDa) in 1-D 8% SDS-PAGE with standard mixture marker proteins. Lane 1-6: Elution fractions after MBP column purification step with a maltose gradient. Lane 7: Elution fraction after superdex elution step. The part of the gel, which is shown in Figure 3, is highlighted in red dashed box.

Figure S3a: *lpg1R*-pMal plasmid construct


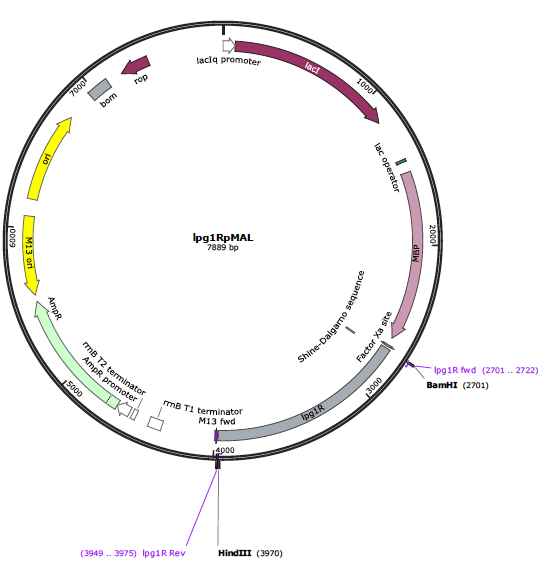


Figure S3b: LPG1R-pMal protein


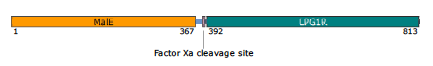


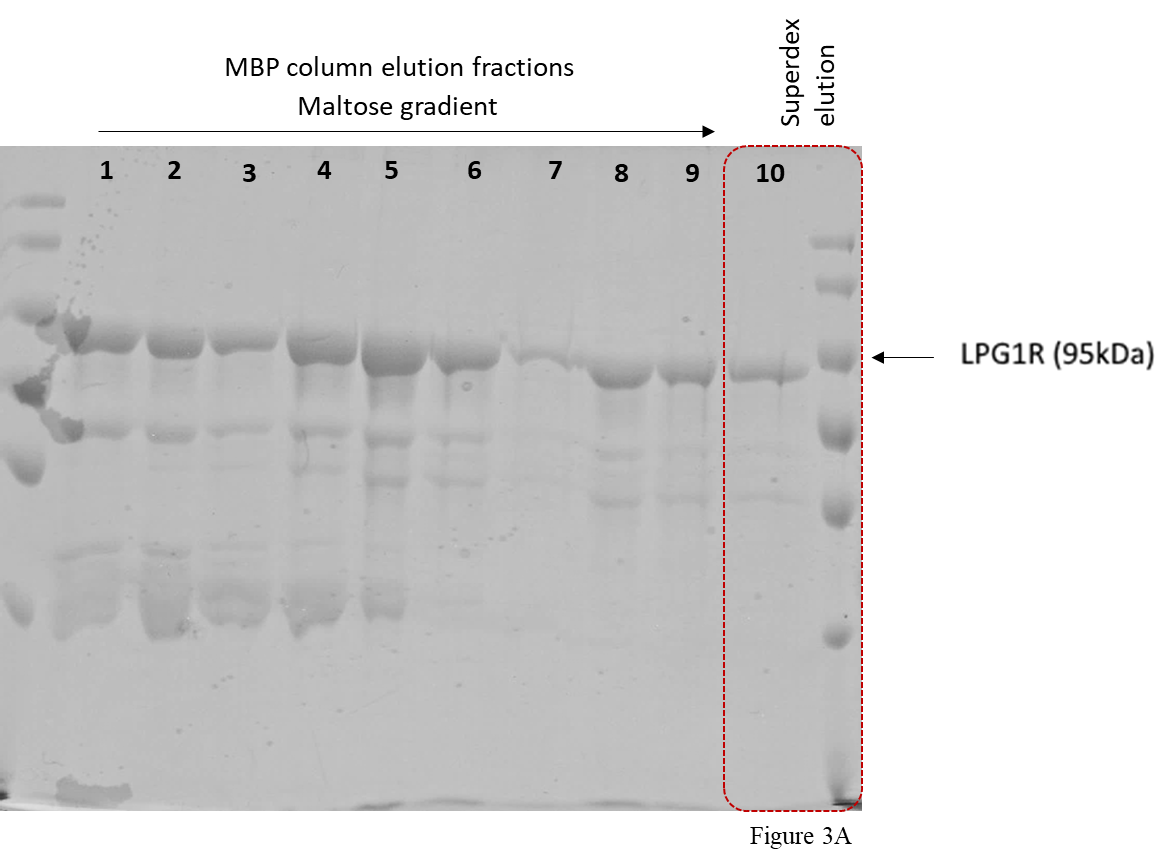


Figure S3c: Evaluation of the expression and the purity of *L. major* LPG1R (95kDa) in 1-D 8% SDS-PAGE with standard mixture marker proteins. Lane 1-9: Elution fractions after MBP column purification step with a maltose gradient. Lane 10: Elution fraction after superdex elution step. The part of the gel, which is shown in Figure 3, is highlighted in red dashed box.

Figure S4a: *lpg1-*pMal plasmid construct


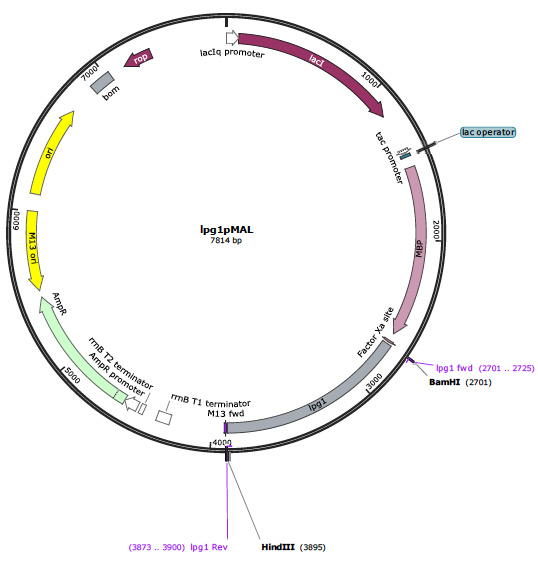


Figure S4b: LPG1-pMal protein


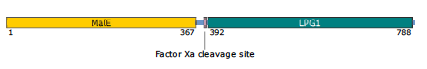


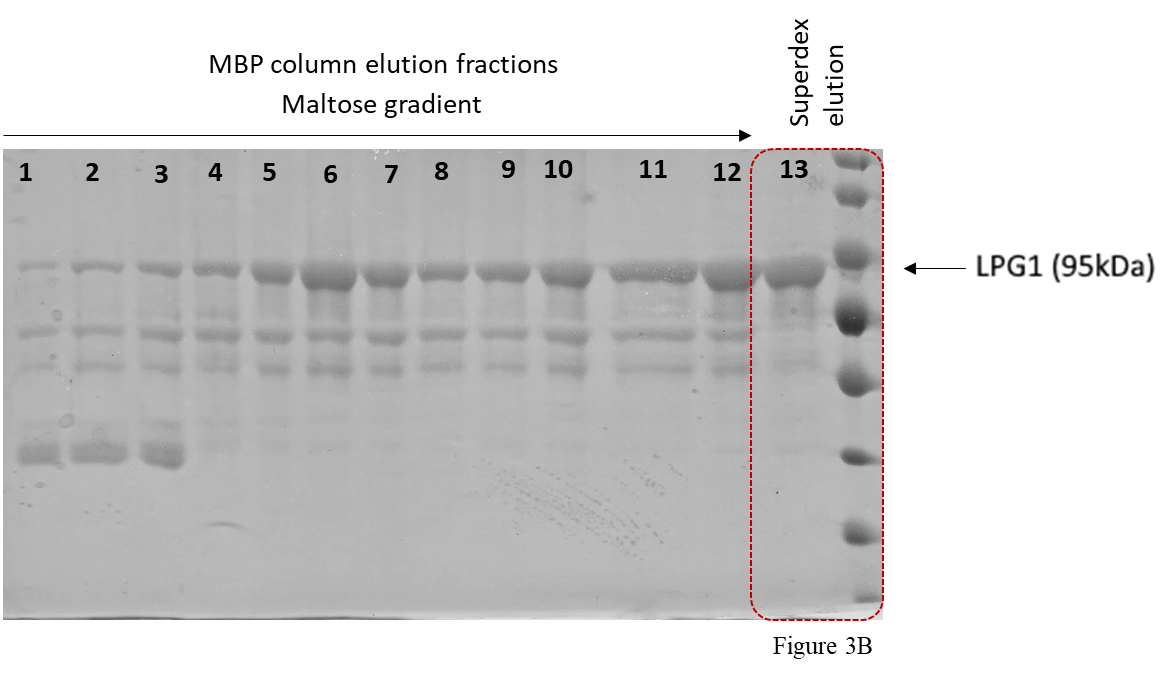


Figure S4c : Evaluation of the expression and the purity of *L. major* LPG1 (95kDa) in 1-D 8% SDS-PAGE with standard mixture marker proteins. Lane 1-12: Elution fractions after MBP column purification step with a maltose gradient. Lane 13: Elution fraction after superdex elution step. The part of the gel, which is shown in Figure 3, is highlighted in red dashed box.

Figure S5: Michaelis-Menten plots for α-D-Methylmannoside glycosylation catalysed by (A) LPG1, (B) LPG1G, (C) LPG1L, and (D) LPG1R in presence of NDP-sugar donors.

**
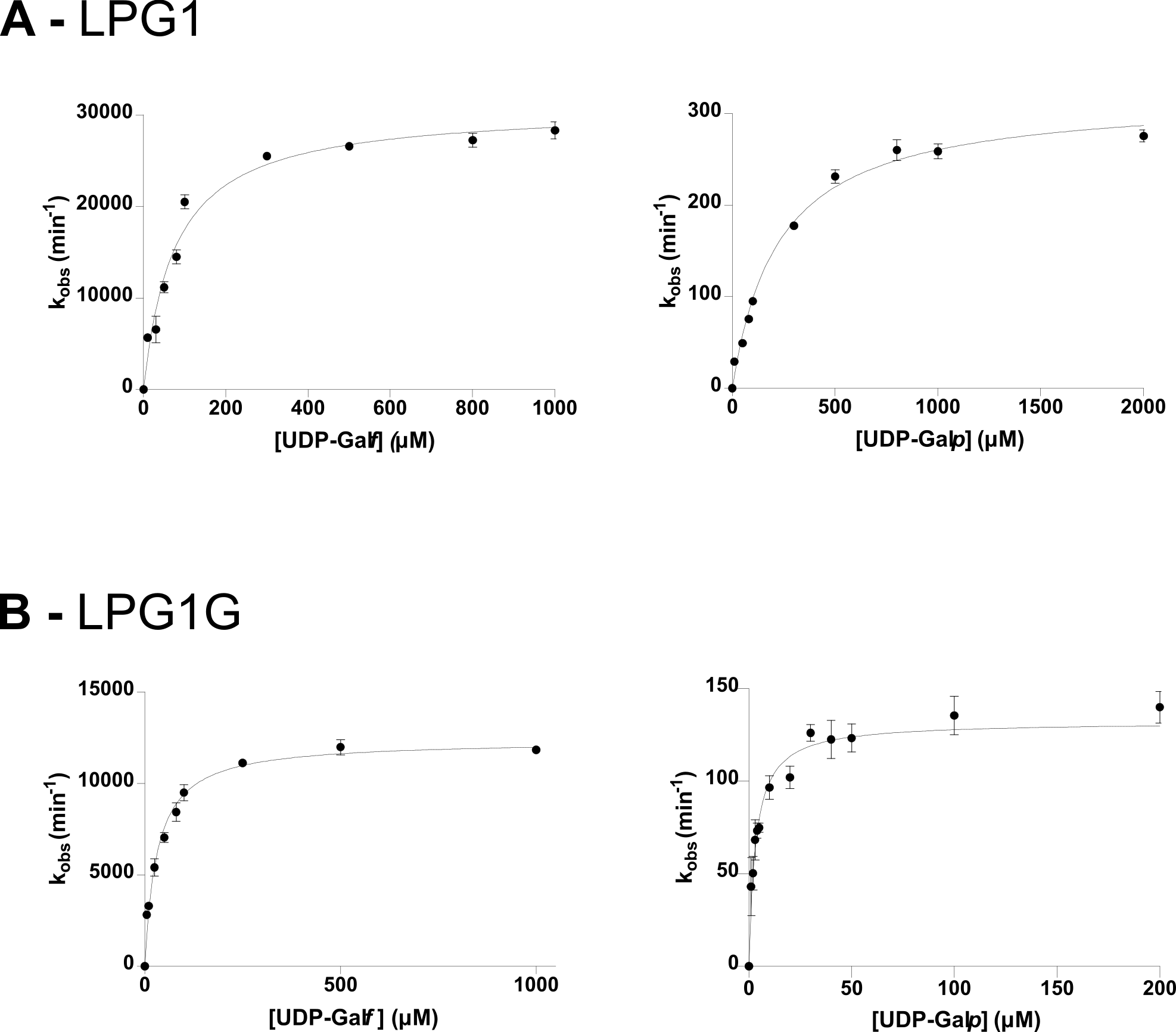
**

**
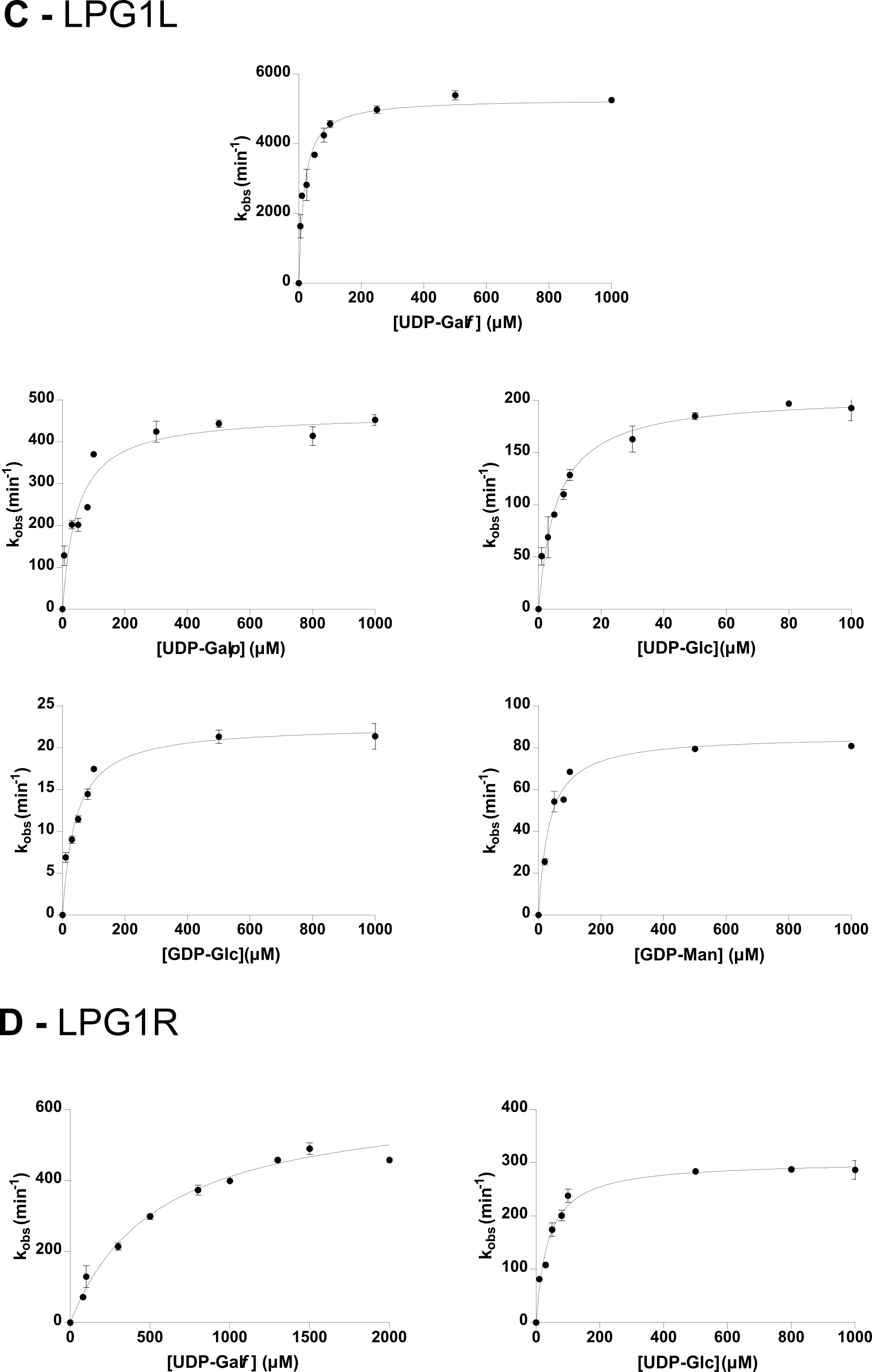
**

Figure S6: Typical TLC plate obtained when analyzing LPG1x-catalyzed transfer of UDP-pyranoses on α-D-Methylmannoside.


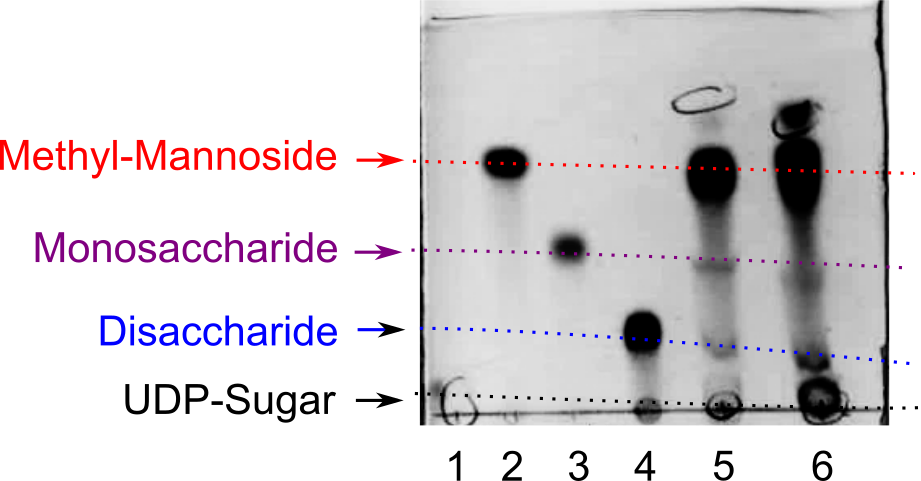


Lane 1 : UDP-Glc*p*

Lane 2 : α-D-Methylmannoside

Lane 3: D-Glucose

Lane 4: Maltose

Lane 5: LPG1G catalysed reaction - UDP-Glc*p* as donor

Lane 6: LPG1G catalysed reaction - UDP-Gal*p* as donor

Figure S7: HRMS analysis of peracetylated disaccharide product after LPG1G-catalyzed reaction using α-D-Methylmannoside as acceptor and (A) UDP-Gal*p* or (B) UDP-Gal*f* as sugar donor. The top panel is a zoomed region of the isolated peak corresponding to disaccharide. The bottom panel is the theoretical spectrum obtained with a chemical formula of C_27_H_38_NaO_18_ (Na adduct of peracetylated dissacharide). No peak was detectable in control reaction (without enzyme or one of the reaction component).

**
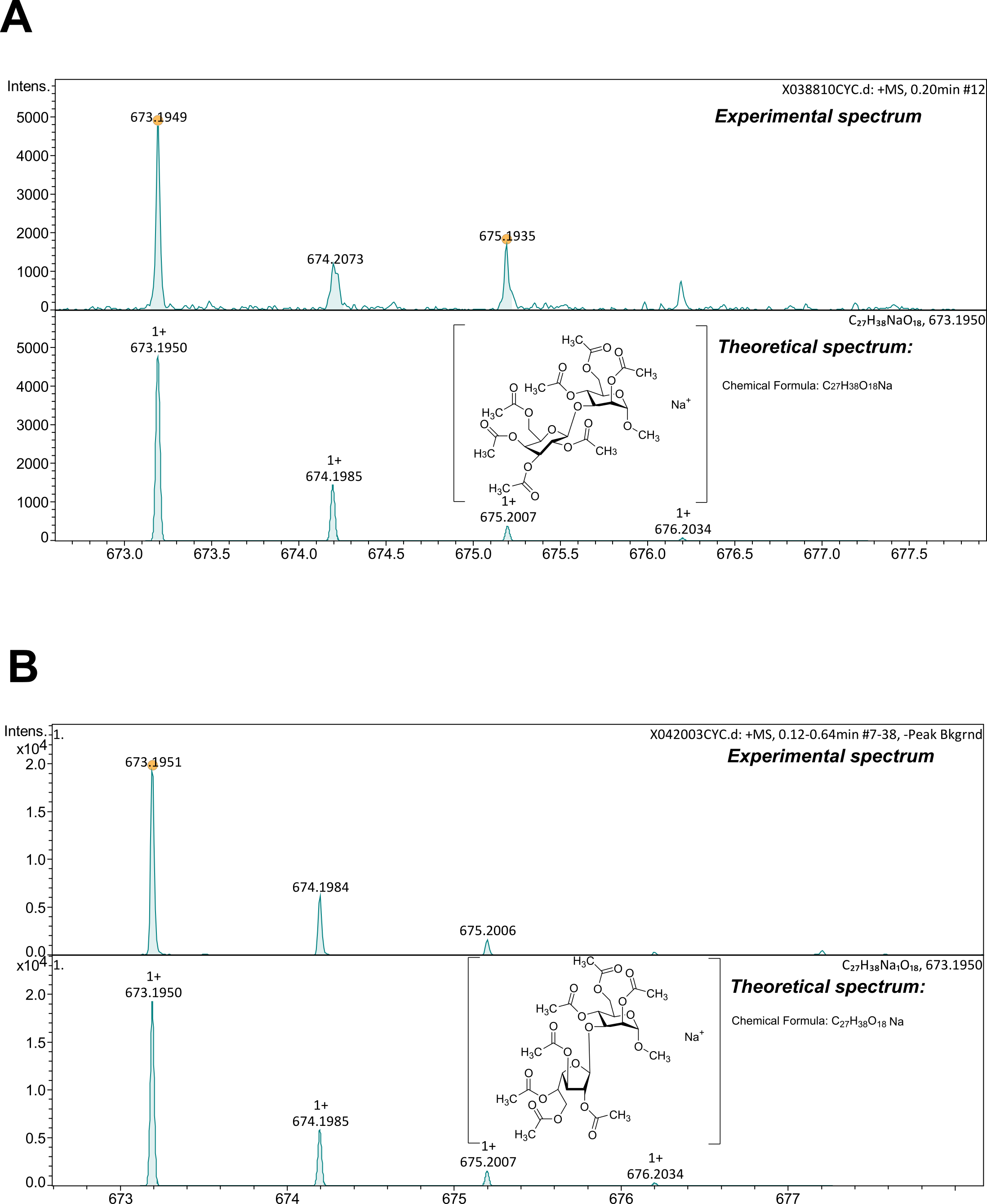
**
